# UHPLC-ESI-MS/MS and GC-MS Analyses on Phenolic, Fatty Acid and Essential Oil of *Verbascum pinetorum* with Antioxidant, Anticholinesterase, Antimicrobial and DNA Damage Protection Effects

**Published:** 2016

**Authors:** Mehmet Boğa, Abdulselam Ertaş, Mustafa Abdullah Yılmaz, Murat Kızıl, Bircan Çeken, Nesrin Haşimi, Tuğba Yılmaz Özden, Serpil Demirci, İsmail Yener, Özcan Deveci

**Affiliations:** a *Department of Pharmaceutical Technology, Faculty of Pharmacy, Dicle University, 21280 Diyarbakır, Turkey. *; b *Department of Pharmacognosy, Faculty of Pharmacy, Dicle University, 21280 Diyarbakır, Turkey*; c *Research and Application of Science and Technology Center (DUBTAM), Dicle University, 21280 Diyarbakir, Turkey. *; d *Department of Chemistry, Faculty of Science and Arts, Dicle University, 21280 Diyarbakır, Turkey. *; e *Department of Nutrition and Dietetics, School of Health, Batman University, 72060 Batman, Turkey.*; f *Department of Biochemistry, Faculty of Pharmacy, Istanbul University, 34116 Istanbul, Turkey.*; g *Department of Pharmaceutical Botany, Faculty of Pharmacy, Cukurova University, 01330 Balcali, Adana, Turkey. *; h *Department of Analytical Chemistry, Faculty of Pharmacy, Dicle University, 21280 Diyarbakır, Turkey.*; i *Department of Infectious diseases and Clinical Microbiology, Faculty of Medicine, Dicle University, 21280, Diyarbakır, Turkey.*

**Keywords:** *Verbascum pinetorum*, fatty acid, essential oil, antioxidant, anticholinesterase, antimicrobial, DNA cleavage protection

## Abstract

This paper is the first phytochemical and ABTS cation radical decolorisation activity, cupric reducing antioxidant capacity, anticholinesterase and DNA damage protection effect of endemic *Verbascum pinetorum *(Boiss.) O. Kuntze. Phenolic profile of *V. pinetorum* were qualified and quantified by UHPLC-ESI-MS/MS analysis. Malic acid (47250.61±2504.28 µg/g) and luteolin (7651.96±527.98 µg/g) were found as most abundant compounds for metanol and acetone extracts, respectively. Fatty acid and essential oil compositions were determined by GC-MS analysis. The main components of fatty acid were found to be palmitic (27.1%) and stearic (22.1%) acids. The main compounds of the essential oil were cineole (16.9%) and α-selinene (16.4%). The acetone extract was found to be more active than BHT used as a standard in β-carotene-linoleic acid test system. In DPPH free radical scavenging activity, the acetone and methanol extracts showed higher activity than BHT at all tested concentrations. The acetone, methanol and water extracts showed strong inhibition while the acetone extract showed better activity than BHT and α-tocopherol which were used as standards in ABTS cation radical scavenging and cupric reducing antioxidant capacity assays, respectively. All extracts were found to be inactive in antialzheimer activity. The acetone extract exhibited moderate antimicrobial activity against *C. albicans. *The methanol extract of *V. pinetorum* were found no significant effect on DNA cleavage protection.

## Introduction

*Verbascum* genus is a member of Scrophulariaceae family and the genus is represented by 271 taxa in which 209 are endemic to Turkey (1-4). *V. pinetorum *(Boiss.) O. Kuntze is 40-100 cm and biannual plant. *V. pinetorum *is endemic to Turkey and is distributed in the East Mediterranean phytogeographical region. It is known in Adana, Hatay and Kahramanmaraş provinces. *V. pinetorum *grows in *Pinus brutia* forest and *Quercus* scrub and altitude between 370-1000 m. Flowering and fructification is between July and August (3). Vernacular name of *V. pinetorum* is Gavurdedengili.

*Verbascum *leaves and flowers are used as mucolytic, expectorant, diuretic and demulcent in traditional Turkish medicine and used to treat respiratory diseases such as tuberculosis, bronchitis, dry coughs, and asthma (5). *Verbascum* species are used as anti-inflammatory on urinary system; and as mild sedatives, and also used for treating rheumatic pain, hemorrhoids, wounds, diarrhea and fungal infections. *Verbascum* species consumed as a tea to relieve abdominal pains (6). *Verbascum *plants have been used for centuries in the folk’s medicine due to their wide spectrum of biological activities. Antimicrobial, antiviral and cytotoxic activities of some *Verbascum *species have been reported elsewhere (7). Some of *Verbascum *species are also used for liquor production (8).

Iridoids and its glycosides (9, 10), flavonoids (11), phenylethanoid and its glycosides (9,11, 12) and neolignan glycosides (12), and saponins (13, 14) have been isolated from *Verbascum* species and some of these compounds have anti-infectious properties and they have antioxidative, antiinflammatory, antitumor and immunostimulatory activities (15).

Synthetic antioxidants are used to extend the shelf life of foods and prevent degradation. However; there are many studies demonstrating the synthetic antioxidants and by-products formed of them can lead to various diseases (16, 17). For this reason, researches about new antioxidant substances in replace of synthetic ones have gained importance in this field. The antioxidants may be also relevant in slowing down the progression of Alzheimer’s disease. So, consumers who want to protect their health have started to deal with natural antioxidants more (18, 19). 

Literature survey showed that there have been no previous reports about phytochemical profile with UHPLC-ESI-MS/MS, fatty acid composition on *Verbascum* species and ABTS cation radical decolorisation activity, cupric reducing antioxidant capacity, anticholinesterase activity and DNA damage effect of* V. pinetorum*. Aim of this study was to determine phytochemical profile, fatty acid and essential oil compositions, antioxidant, antialzheimer, antimicrobial activities and DNA cleavage protection of the extracts of endemic *V. pinetorum *from Turkey. This study is the first phytochemical and biological (ABTS, Cuprac, anticholinesterase and DNA damage protection effect) report on *V. pinetorum*.

## Experimental

*General experimental procedures*

Phytochemical profile, essential oil and fatty acid compositions were determined by using Shimadzu UHPLC ESI MS/MS and GC/MS instruments, respectively. A Thermo pH-meter, Gel documentation System (Gel-Doc-XR, BioRad, Hercules, CA, USA), an Elma S15 ultrasonic bath,Horizontal electrophoresis (Biorad), Horizontal electrophoresis power supply (Wealtec), Shimadzu UV Spectrophotometer, a BioTek Power Wave XS and a vortex (LMS Co. LTD) were used for the activity assays. Ethanol, hexane, diethyl ether, chloroform, toluene, dichloromethane, methanol, potassium acetate, BHT (butylated hydroxytoluene) (purity ≥99%), sulphuric acid, aluminium nitrate nonahydrate, aluminium chloride, ABTS (2,2′-Azinobis (3-ethylbenzothiazoline-6-sulfonic acid) diammonium salt) (97.5%), K_2_S_2_O_8_, sodium acetate, nutrient broth, boric acid, nutrient agar, NaHCO_3_ were purchased from Merck (Germany), (L)-malic acid (95-100%), quercetin (95%), protocatechuic acid (97%), chrysin (97%), rutin (94%), hesperetin (95%), naringenin (95%), rosmarinic acid (96%), vanillin (99%), p-coumaric acid (98%), caffeic acid (98%), chlorogenic acid (95%), hyperoside (≥97%), myricetin (≥96%), coumarin (≥99%), kaempferol (≥97%), 2,2-diphenyl-1-picrylhydrazyl (DPPH) (≥95%), *β*-carotene (≥93%), linoleic acid (≥99%), H_2_O_2_, Tween 40, pyrocathecol (≥99%), acetic acid, sodium methoxide, gel loading dye, DTNB (5,5-dithiobis-(2-nitro benzoic acid)) (≥98%), copper (II) chloride dihydrate (CuCl_2_.2H_2_O) (≥99%), neocuproine (2,9-dimethyl-1,10-phenanthroline) (≥98%), EDTA (≥98%), acetylcholinesterase, butyrylcholinesterase, trisma base, galanthamine hydrobromide (≥94%) from Sigma (Germany), α-tocopherol (≥95.5%), acetylthiocholine iodide (≥98%) from Aldrich (Germany), quinic acid (98%), tr-aconitic acid (98%), 4-hydroxybenzoic acid (≥99%) and fisetin (≥98%) were from Aldrich (Germany), gallic acid (≥99%), tannic acid (puris), salicylic acid (≥99%) were from Sigma-Aldrich (Germany), Folin Ciocalteu Phenol reagent from Applichem (Germany), hesperidin (≥97%), luteolin (≥97%), apigenin (≥99%), rhamnetin (≥99%), butyrylthiocholine iodide (≥99%) from Fluka (Germany), steril blank disc and antbiotic disc from Oxoid (United Kingdom), acetone, petroleum ether,  sodium carbonate, sodium dihydrogen phosphate, sodium hydrogen phosphate, ammonium acetate from Reidel de Haen (Germany).

*Plant material*

S. Demirci collected and identified the plant material from southern Turkey (Andırın, Kahramanmaraş) in June 2012. A sample was deposited in the Herbarium of Istanbul University (ISTE 97137). 

*Preparation of plant extracts for UHPLC-ESI-MS/MS*

The dried and powdered plants (10 g) were extracted separately with MeOH and acetone about 24 h at room temperature. The extracts were filtrated and evaporated under vacuum. Dry filtrates diluted until 250 mg/L and passed through 0.2 µM microfiber filter for UHPLC-ESI-MS/MS.

*Instruments and Chromatographic Conditions*

LC-MS/MS analyses of the twenty seven compounds were performed by using a Nexera model Shimadzu UHPLC coupled to a tandem MS instrument. The liquid chromatograph was equipped with LC-30AD binary pumps, DGU-20A3R degasser, CTO-10ASvp column oven and SIL-30AC autosampler. The chromatographic seperation was performed on a C18 reversed-phase Inertsil ODS-4 (150 mm × 4.6 mm, 3*µ*M) analytical column. The column temperature was fixed at 40 ° C. The elution gradient consisted of mobile phase A (water, 5mM ammonium formate and 0.1% formic acid) and mobile phase B (methanol, 5mM ammonium formate and 0.1% formic acid). The gradient program with the following proportions of solvent B was applied t (min), %B: (0, 40), (20, 90), (23.99, 90), (24, 40), (29, 40). The solvent flow rate was maintained at 0.5 mL/min and injection volume was settled as 4 *µ*L.

*MS Instrumentation*

MS detection was performed using Shimadzu LC-MS 8040 model triple quadrupole mass spectrometer equipped with an ESI source operating in both positive and negative ionization modes. LC-MS/MS data were collected and processed by LabSolutions software (Shimadzu, Kyoto, Japan). The multiple reaction monitoring (MRM) mode was used to quantify the analytes: the assay of investigated compounds was performed following two or three transitions per compound, the first one for quantitative purposes and the second and/or the third one for confirmation.

*Optimization of UHPLC-MS/MS Method*

Subsequent to several combinations of trials, a gradient of methanol (5 mM ammonium formate and 0.1% formic acid) and water (5mM ammonium formate and 0.1% formic acid) system was concluded to be the best mobile phase solution. For rich ionization and the seperation of the molecules, the mentioned mobile phase was proved to be the best of all. ESI source was chosen instead of APCI (Atmospheric Pressure Chemical Ionization) and APPI (Atmospheric Pressure Photoionization) sources as the phenolic compounds were small and relatively polar molecules. Tandem mass spectrometry was decided to be used for the current study since this system is commonly used for its fragmented ion stability. The working conditions were determined as interface temperature; 350 ° C, DL temperature; 250 ° C, heat block temperature; 400 ° C, nebulizing gas flow (Nitrogen); 3 L/min and drying gas flow (Nitrogen); 15 L/min.

*Esterification of total fatty acids and GC/MS conditions*

Esterification of petroleum ether extract of *V. pinetorum* was done according to the report of Ertaş *et al.* (19). In this study, Thermo Scientific Polaris Q GC-MS/MS was used. GC-MS study conditions, identification and quantification of the compounds comparison were done exactly same manner according to Ertaş *et al.* (19).

*Isolation of essential oil and GC/MS conditions*

Clevenger-type apparatus was used for obtaining essential oil of *V. pinetorum. *The essential oil was diluted for the GC process by dichloromethane (1:3, v/v). GC/MS analyses were performed on Thermo Electron Trace 2000 GC model gas chromatography and Thermo Electron DSQ quadrupole mass spectrometry. A nonpolar Phenomenex DB5 fused silica column (30 m´ 0.32 mm, 0.25 μM film thickness) was used with helium at 1mL/min (20 psi) as a carrier gas. The GC oven temperature was kept at 60 ° C for 10 min and programmed to 280 ° C for 10 min. The split ratio was adjusted to 1:50, the injection volume was 0.1 μL and EI/MS was recorded at 70eV ionization energy. Mass range was *m/z*35-500 amu. Identification of the compounds was based on the comparison of their retention times and mass spectra with those obtained from authentic samples and/or the NIST and Wiley spectra as well as the literature data.

*Preparation of the extracts*

Whole parts of *V. pinetorum *(100 g) were dried under shadow and powdered, and then they were sequentially macerated 3 times with petroleum ether, acetone, methanol and water (250 mL) for 24 h at room temperature, respectively. After filtration, the solvents were evaporated to obtain crude extracts. The yield of the extracts are petroleum ether extract 0.60%, acetone extract 1.20%, methanol extract 5.00% and water extract 2.30% (w/w).

*Determination of total phenolic and flavonoid contents of the extracts*

The amounts of phenolic and flavonoid contents in the crude extracts were expressed as pyrocatechol and quercetin equivalents, and they were calculated according to the following equations (20, 21):

Absorbance = 0.0125 pyrocatechol (μg) + 0.0347 (R^2 ^= 0.9928)

 Absorbance = 0.0301 quercetin (μg) + 0.0553 (R^2 ^= 0.9984)

*Antioxidant activity of the extracts*

*β*-Carotene-linoleic acid test system (22), DPPH free radical scavenging activity (23), ABTS cation radical decolorisation (24) and cupric reducing antioxidant capacity (CUPRAC) (25) methods were carried out to determine the antioxidant activity.

*Anticholinesterase activity of the extracts*

A spectrophotometric method developed by Ellman et al. was established to indicate the acetyl- and butyryl-cholinesterase inhibitory activities (26).

*Determination of antimicrobial activity and Minimum Inhibitory concentration (MIC)*

Five different microorganisms including gram positive bacteria (*Streptococcus pyogenes *ATCC19615 and *Staphylococcus aureus* ATCC 25923), gram negative bacteria (*Pseudomonas aeruginosa* ATCC 27853, *Escherichia coli* ATCC 25922) and yeast (*Candida albicans *ATCC10231) which were purchased from Refik Saydam Sanitation Center (Turkey) were used for detecting the antimicrobial activity of the samples. The disc diffusion method was employed for this purpose (27, 28). The minimum inhibitory concentration determined by the broth macrodilution method according to NCCLS (29). Ampicillin and fluconazole were used as positive controls for bacteria and yeast, respectively.

*DNA damage protective activity of the extracts*

Measurement of the DNA damage protective activity of the methanol extract was checked on pBluescript M13(+) plasmid DNA. Plasmid DNA was oxidized with OH radicals which generated from UV photholysis of H_2_O_2_ in the presence of theextract and checked on 1% agarose according to Kızıl *et al.* (30). Percent inhibition of the DNA cleavage protection was calculated using the method described by Fukuhara *et al*. (31).

*Statistical analysis*

The results of the antioxidant, anticholinesterase and antimicrobial activity assays were mean ± SD of three parallel measurements. The statistical significance was estimated using a Student’s *t*-test, *p* values *<*0.05 were regarded as significant.

**Figure 1:**
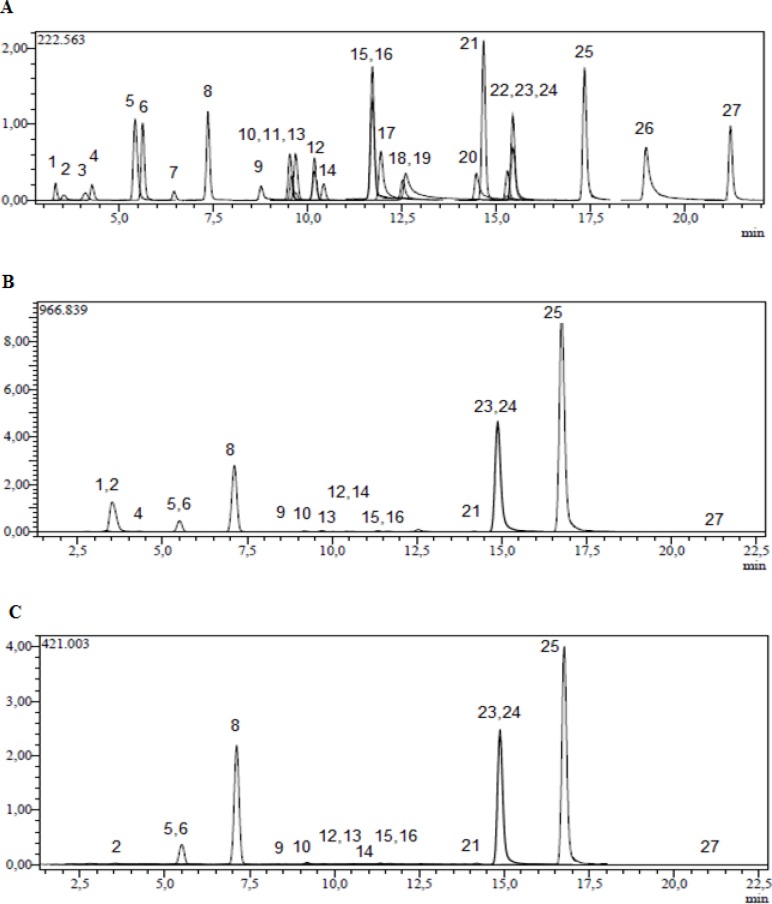
UHPLC ESI-MS/MS chromatograms of A: 250 ppb standard mix, B: methanol extract of *V. pinetorum* C: acetone extract of *V. pinetorum*

**Figure 2:**
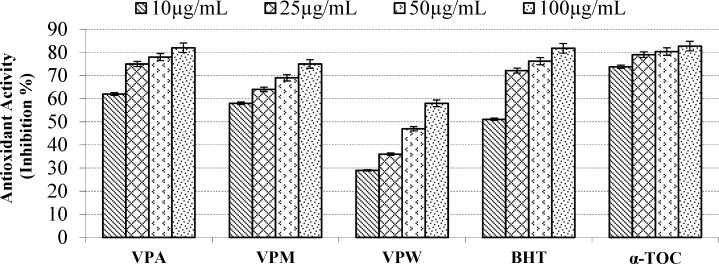
Inhibition (%) of lipid peroxidation of the extracts, BHT and α-TOC by β-carotene bleaching method. Values are means ±S.D. of three parallel measurements

**Figure 3:**
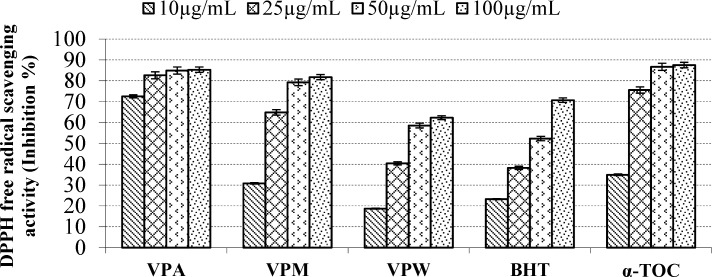
Free radical scavenging activity of the extracts, BHT and α-TOC. Values are means ±S.D. of three parallel measurements

**Figure 4:**
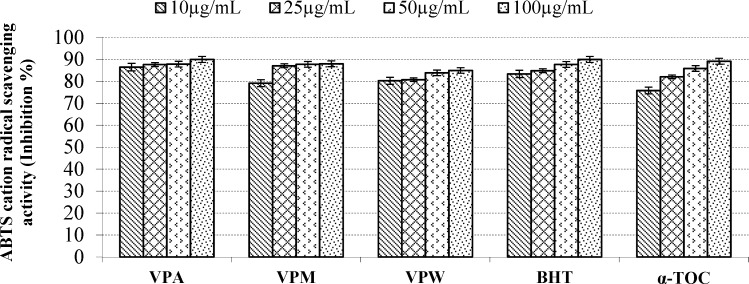
ABTS cation radical scavenging activity of the extracts, BHT and α-TOC. Values are means ±S.D. of three parallel measurements

**Figure 5:**
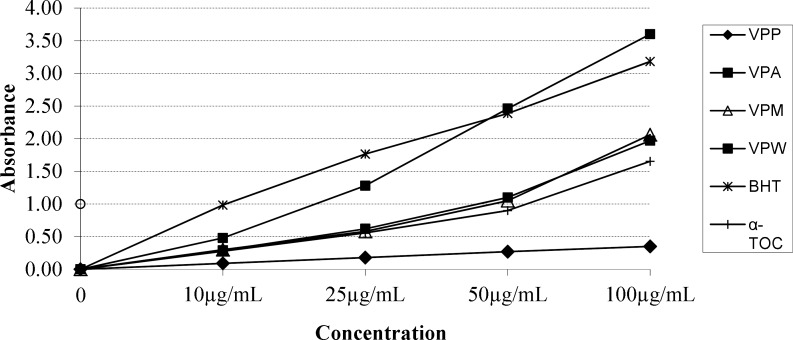
Cupric reducing antioxidant capacity of the extracts, BHT and α-TOC. Values are means ±S.D. of three parallel measurements

**Figure 6:**
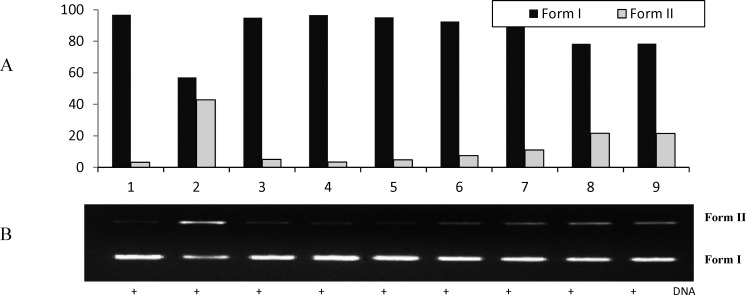
The quantified band intensity for the scDNA (Form I), ocDNA (Form II) with Quantity One 4.5.2. version software (A). Electrophoretic pattern of pBluescript M13+ DNA after UV-photolysis of H_2_O_2_ in the presence or absence of *V. pinetorum* methanol extract*. *Reaction vials contained 200 ng of supercoiled DNA (31.53 nM) in distilled water, pH 7). Electrophoresis was performed using 1% agarose at 40V for 3 h in the presence of ethidium bromide (10 mg/mL) (B). Electrophoresis running buffer: TAE (40 mM Tris acetate, 1 mM EDTA, pH 8.2). Gel was scanned on Gel documentation system (Gel-Doc-XR, BioRad, Hercules, CA, USA). Bands on the gels were quantified using discovery series Quantity One programme (version 4.5.2. BioRad Co

**Table 1.** Analytical parameters of the UHPLC-ESI-MS/MS method and identification and quantification of phenolic compounds of *V. pinetorum* methanol and acetone extracts by UHPLC-ESI-MS/MS^a^

No	Analyte	Parent ion (m/z)^a^	MS^2^(CE)^b^	Ionization Mode	RT^c^	R^2,^^d^	RSD%^e^	Linearity Range (mg/L)	LOD/LOQ (µg/L)^f^	Recovery (%)
1	Quinic acid	190,95	85 (22),93 (22)	Neg	3.32	0.9927	0.0388	250-10000	22.3 / 74.5	103.3
2	Malic acid	133,05	115 (14),71 (17)	Neg	3.54	0.9975	0.1214	250-10000	19.2 / 64.1	101.4
3	tr-Aconitic acid	172,85	85 (12),129 (9)	Neg	4.13	0.9933	0.3908	250-10000	15.6 / 51.9	102.8
4	Gallic acid	169,05	125 (14),79 (25)	Neg	4.29	0.9901	0.4734	25-1000	4.8 / 15.9	102.3
5	Chlorogenic acid	353	191 (17)	Neg	5.43	0.9932	0.1882	250-10000	7.3 / 24.3	99.7
6	Protocatechuic acid	152,95	109 (16),108 (26)	Neg	5.63	0.9991	0.5958	100-4000	25.8 / 85.9	100.2
7	Tannic acid	182,95	124 (22),78 (34)	Neg	6.46	0.9955	0.9075	100-4000	10.2 / 34.2	97.8
8	tr- caffeic acid	178,95	135 (15),134 (24),89 (31)	Neg	7.37	0.9942	1.0080	25-1000	4.4 / 14.7	98.6
9	Vanillin	151,05	136 (17),92 (21)	Neg	8.77	0.9995	0.4094	250-10000	10.1 / 33.7	99.2
10	p-Coumaric acid	162,95	119 (15),93 (31)	Neg	9.53	0.9909	1.1358	100-4000	15.2 / 50.8	98.4
11	Rosmarinic acid	358,9	161 (17),133 (42)	Neg	9.57	0.9992	0.5220	250-10000	10.4 / 34.8	101.7
12	Rutin	609,1	300 (37), 271 (51), 301 (38)	Neg	10.18	0.9971	0.8146	250-10000	17.0 / 56.6	102.2
13	Hesperidin	611,1	303 (24),465 (12)	Poz	9.69	0.9973	0.1363	250-10000	21.6 / 71.9	100.2
14	Hyperoside	463,1	300 (27),301 (26)	Neg	10.43	0.9549	0.2135	100-4000	12.4 / 41.4	98.5
15	4-OH Benzoic acid	136,95	93 (17),65 (27)	Neg	11.72	0.9925	1.4013	25-1000	3.0 / 10.0	106.2
16	Salicylic acid	136,95	93 (16),65 (31),75 (30)	Neg	11.72	0.9904	0.6619	25-1000	4 / 13.3	106.2
17	Myricetin	317	179 (19),151 (23),137 (26)	Neg	11.94	0.9991	2.8247	100-4000	9.9 / 32.9	106.0
18	Fisetin	284,95	135 (22),121 (27)	Neg	12.61	0.9988	2.4262	100-4000	10.7 / 35.6	96.9
19	Coumarin	146,95	103 (17),91 (26),77 (27)	Poz	12.52	0.9924	0.4203	100-4000	9.1 / 30.4	104.4
20	Quercetin	300,9	179 (19),151 (21),121 (28)	Neg	14.48	0.9995	4.3149	25-1000	2.0 / 6.8	98.9
21	Naringenin	270,95	151 (18),119 (24),107 (26)	Neg	14.66	0.9956	2.0200	25-1000	2.6 / 8.8	97.0
22	Hesperetin	300,95	164 (25),136 (33),108 (42)	Neg	15.29	0.9961	1.0164	25-1000	3.3/ 11.0	102.4
23	Luteolin	284,95	217 (25),199 (28),175 (29),151 (25)	Neg	15.43	0.9992	3.9487	25-1000	5.8 / 19.4	105.4
24	Kaempferol	284,95	217 (29),133 (32),151 (23)	Neg	15.43	0.9917	0.5885	25-1000	2.0 / 6.6	99.1
25	Apigenin	268,95	151 (25),117 (35)	Neg	17.31	0.9954	0.6782	25-1000	0.1 / 0.3	98.9
26	Rhamnetin	314,95	165 (23),121 (28),300 (22)	Neg	18.94	0.9994	2.5678	25-1000	0.2 / 0.7	100.8
27	Chrysin	253	143 (29),119 (32),107 (26)	Neg	21.18	0.9965	1.5530	25-1000	0.05 / 0.17	102.2

a Parent ion (*m/z):* Molecular ions of the standard compounds (mass to charge ratio)

b MS^2^(CE): MRM fragments for the related molecular ions (CE refers to related collision energies of the fragment ions)

c RT: Retention time

d R^2^: coefficient of determination

e R^2^: coefficient of determination

f LOD/LOQ (µg/L): Limit of deteection/Limit of quantification

g U (%): Percent relative uncertainty at 95% confidence level (k=2).

h Values in *µ*g/g (w/w) of plant extract

i N.D: not detected

**Table 2:** Fatty acid analysis of *V. pinetorum* petroleum ether extract

**Rt (min)** a	**Constituents** b	**% Composition**
14.39	10-Undecenoic acid	0.4
18.60	Myristic acid	0.6
25.27	Palmitic acid	27.1
30.64	Linoleic acid	17.1
30.77	Oleic acid	11.8
30.86	Linolenic acid	15.1
31.54	Stearic acid	22.1
37.38	Arachidic acid	1.5
39.36	Docosane	4.1
	Total	99.8

a Retention time (as minutes)

b A nonpolar Phenomenex DB-5 fused silica colum

**Table 3.** Essential oil composition of *V. pinetorum*

**Rt (min)** a	**Constituents** b	**% Composition**	**RI** c
10.87	Isononane	2.6	865
15.20	β-pinene	2.3	979
17.15	Cineole	16.9	1031
24.06	1,3-Di-tert butyl benzene	3.2	1249
25.80	Dihydro carvyl acetate	3.5	1344
30.30	τ-Muurolene	3.4	1480
30.48	Valencene	2.7	1484
30.87	α-Selinene	16.4	1498
35.52	2-Methyl heptadecane	2.8	1746
36.45	Octadecane	2.9	1800
36.74	2-Methyl-1-hexadecanol	2.4	1890
36.93	1-Nonadecanol	2.8	2156
40.00	Heneicosane	3.1	2109
40.13	2,5-Di-tert octyl-p-benzoquinone	7.8	2259
40.59	Arachidic acid	3.5	2366
40.66	Hexadecanoic acid	2.4	1986
40.84	Tetracosane	2.4	2407
41.13	3-Ethyl-5-(2-ethylbutyl)octadecane	2.8	2413
43.30	Heptacosane	3.1	2700
43.84	Choleic acid	2.9	2896
44.41	Ethyl iso-allocholate	2.3	3094
45.11	17-pentatriacontene	2.6	3508
46.50	Hexatriacontane	2.3	3600
47.12	Tetratetracontane	2.4	4400
	Total	99.5	

a Retention time (as minutes).

b A nonpolar Phenomenex DB-5 fused silica column

c RI Retention indices (DB-5 column)

**Table 4:** Total phenolic and flavonoid contents and anticholinesterase activity of *V. pinetorum* extracts^x^

**Extracts**	**Phenolic content** **(μg PEs/mg extract)** z	**Flavonoid content** **(μgQEs/mg extract)** t	**Inhibition %** **against AChE**	**Inhibition %** **against BChE**
VPP	139.20 ± 2.83	92.09 ± 1.38	NA	44.02 ± 0.98
VPA	577.20 ± 2.63	111.03 ± 1.21	NA	15.64 ± 0.56
VPM	293.11 ± 1.31	27.97 ± 0.33	NA	25.05±0.19
VPW	339.42 ± 1.11	74.15 ± 0.23	NA	11.14±0.72
Galanthaminey	-	-	75.11 ± 0.69	82.49 ± 0.32

x Values expressed are means ± S.D. of three parallel measurements, different letters in the same column indicate a significant difference (*p*< 0.05),

y Standard drug, NA: Not active,

z PEs, pyrocatechol equivalents (y = 0.0125 x + 0.0347 R^2 ^= 0.9928),

t QEs, quercetin equivalents (y = 0.0301 x – 0.0553 R^2 ^= 0.9984).

**Table 5:** Zones of growth inhibition (mm) and MIC values showing the antimicrobial activities of the extracts compared to positive controls.

***Microorganisms ***											
		***Gram positive***				***Gram negative***				***Yeast***	
			***S. aureus***		***S.pyogenes***		***E.coli***		***P. aeruginosa***		***C. albicans***	
Acetone extract	^a^DD	8±0.6		10±0.2		10±0.3		-		16±0.2	
	MIC	85±0.2		>1000		>1000		-		25±0.3	
Methanol extract	aDD	8±0.3		8±0.5		10±0.2		10±0.3		-	
	MIC	40±0.5		>1000		>1000		>1000		-	
Water extract	^a^DD	10±0.3		10±0.3		NZ		10±0.2		-	
	MIC	30±0.2		>1000		-		>1000		-	
Positive controls	bDD	35±0.2		19±0.2		20±0.1		-		30±0.3	
	MIC	1.95±0.3		7.815±0.1		7.815±0.4		-		3.125±0.2	

-: Not active

a DD: Inhibition zone in diameter (mm) around the discs (6 mm) impregnated with 30 mg mL^-1^ of plant extracts.

b DD: Inhibition zone in diameter (mm) of positive controls that are ampicillin for bacteria and fluconazole for yeast. Minimum inhibitory concentration (MIC) values are given as μg mL^-1^

## Results and discussion

*Quantitative analysis of phenolic compounds by UHPLC-ESI (QqQ)/MS/MS*

Several studies are present in literature reporting the use of liquid chromatography electrospray ionization tandem mass spectrometry to perform quantitative analyses (32-34). Thus, for quantitative purpose, an accurate method on a mass spectrometer equipped with a triple quadrupole analyzer was developed for the analyses of twenty-seven compounds. Methanol and acetone extracts of *V. pinetorum *were analysed in order to quantify the twenty seven compounds.

In order to monitor the mentioned compounds by MRM, the specific fragmentation reactions were selected. Twenty-seven compounds including (L)-malic acid, quercetin, protocatechuic acid, chrysin, rutin, hesperetin, naringenin, rosmarinic acid, vanillin, p-coumaric acid, caffeic acid, chlorogenic acid, hyperoside, myricetin, coumarin, kaempferol, quinic acid, tr-aconitic acid, 4-hydroxybenzoic acid, fisetin, gallic acid, tannic acid, salicylic acid, hesperidin, luteolin, apigenin, rhamnetin were monitored by the transition from the specific deprotonated molecular ions [M-H^+^] to the corresponding fragment ions [M-H^+^-X] and Figure 1A shows their chromatograms. Table 1. shows molecular ions, fragments observed in MS/MS, related collision energies for these fragments and the quantified results for methanol and acetone extracts of *V. pinetorum*.

For metanol and acetone extract of *V. pinetorum*; tr-aconitic acid, tannic acid, rosmarinic acid, myricetin, fisetin, coumarin, quercetin, hesperetin, rhamnetin compounds and for acetone extract of *V. pinetorum*; quinic acid, gallic acid were not detected and quantified by this method. In the methanol extract of *V. pinetorum,* malic acid (47250.61 µg/g), apigenin (16875.45 µg/g), kaempferol (16834.42 µg/g) and luteolin (15693.14 µg/g) were found to be major compounds (Table 1, Figure 1B) and for acetone extract of *V. pinetorum, *luteolin (7651.96 µg/g), kaempferol (7552.63 µg/g) and apigenin (7159.58 µg/g) were the most abundant compounds (Table 1., Figure 1C.). In literature several phenolic compounds such as chlorogenic acid, caffeic acid, ferulic acid, rosmarinic acid, quercetin, apigenin and aucubin from *V. phlomoides* (35), luteolin, apigenin, diosmin and 7-glucosides of luteolin, apigenin and quercetin from *V. densiflorum* and *V. phlomoides* (11), caffeic, syringic, p-coumaric, ferulic acid, quercetin and rutin from *V. pestalozzae* (36), protocatechuic, chlorogenic, vanillic, p-coumaric acid and rutin from *V. detersile* (36), protocatechuic, chlorogenic, p-coumaric acid, quercetin and rutin from *V. bellum* (36), protocatechuic, chlorogenic, caffeic, p-coumaric, ferulic acid and rutin from *V. myriocarpum* (36) have been detected in *Verbascum* species by HPLC DAD technique. In literature, there is not any study about qualitative and quantitative phenolic and flavonoid constituents on *Verbascum *species with UHPLC-MS/MS instrument. 

There is no report about fatty acid composition on *Verbascum *species and also essential oil composition studies on *V. pinetorum*. This is the first study which report fatty acid and essential oil composition of *V. pinetorum*. As shown in Table 2. fatty acid composition of petroleum ether extract of *V. pinetorum *was determined by GC and GC-MS analysis and 9 components were identified, constituting 99.8%. The main components of fatty acid were found to be palmitic (27.1%), stearic (22.1%) and linoleic acids (17.1%). In the study of Yılmaz-İskender *et al*. (37) flower, leaf and stem of *V. wiedemannianum* were analysed for their volatile components. The main group of contituents of oil composition of flower and stem is hydrocarbons with 83.3% and 32.1% ratios, respectively. Major group of leaf oil was found to be aldehydes with 46.8% ratio. Main components of flower, leaf and stem oils were pentadecane (58.2%), (2E)-hexanal (33.2%) and hexadecanoic acid (24.6%), respectively.

There is no report about essential oil composition of *V. pinetorum*. The essential oil composition of *V. pinetorum* were determined by GC and GC-MS analysis. 24 compounds were identified, constituting 99.5% of total oil (Table 3.). The main compounds of the essential oil were cineole (16.9%) and α-selinene (16.4%). According to the report of Melliou *et al*. (38) thirty-six compounds were identified from essential oil of *V. undulatum* Lam and the main compounds were 1-octen-3-ol (22.5%) and α-bisabolol (10.6%). The antioxidant activity of the petroleum ether (VPP), acetone (VPA), methanol (VPM) and water (VPW) extracts prepared from the whole plant of *V. pinetorum* was investigated by using *β*-carotene bleaching, DPPH free radical scavenging, ABTS cation radical decolorisation and cupric reducing antioxidant capacity assays with their total phenolic and flavonoid contents.

According to report of Alan *et al*. (39) on the antioxidant activities of three *Verbascum *species, employing different extraction solvents, the methanol and water extracts exerted greater antioxidant activity than other extracts. Results of the study of Saltan *et al*. (36) indicate that the methanol extract of various *Verbascum* species showed good antioxidant acitivty in β-carotene-linoleic acid test system and DPPH free radical scavenging assays. In β-carotene-linoleic acid test system, the acetone extract of *V. pinetorum *showed better inhibition than BHT at all concentrations (Figure 2.) in present study. The acetone and methanol extracts of *V. pinetorum* showed strong inhibition in DPPH free radical scavenging and have found to be higher than BHT at all concentration (Figure 3.). The acetone, methanol and water extracts of *V. pinetorum *exhibited significant inhibition in ABTS cation radical scavenging assay and also the acetone extract of *V. pinetorum* is more active than BHT and α-Toc used as standards (Figure 4.). In CUPRAC reducing antioxidant capacity assay, the acetone, methanol and water extracts showed strong activity; while the activity of the acetone extract was even better than standards BHT and α-Toc. Also the methanol and water extracts showed higher activity than the standard α-Toc (Figure 5.). As seen in Table 4., total phenolic and flavonoid contens of acetone extract were the richest and total phenolic content is more than total flavonoid content for all extracts. There is no activity against acethylcholinesterase and weak activity against butyrylcholinesterase extracts of *V. pinetorum* in antialzheimer activity assays (Table 4.).

The antimicrobial activities of the extracts against different microorganisms were assessed according to inhibition zone diameter and MIC value. Results are presented in Table 5. In reports of Saltan *et al*. (36), generally ethylacetate extracts were effective for *E. coli. *Ethylacetate extract of *V. pestalozzae *showed the best inhibitory effect on *P. aeroginosa*. The antimicrobial activity of *V. pinetorum.*extracts against different microorganisms were assessed according to inhibition zone diameter. Among the extracts only the petroleum ether extract was not active on microorganisms (data not shown). The acetone, methanol and water extracts showed activity in different degrees. The acetone extract showed weak antimicrobial activity (inhibition zone < 12) against *E. coli*, *S. pyogenes*, *S. aureus*; moderate activity (inhibition zone < 20-12) against *C. albicans*; and no activity against *P. aeruginosa*. The methanol extract showed weak antimicrobial activity against bacteria and no activity against the yeast. The water extract was active only on *P. aeruginosa*,* S. pyogenes* and *S. aureus*. The lowest MIC value was recorded by acetone extract against *C. albicans* (25 µg mL^-1^).

The inhibition activities of the methanol extract of *V. pinetorum* on DNA damage were found to be 89.34, 80.31, 53.91 and 53.68 % at the concentrations of 100, 250, 350 and 500 μg/mL, respectively. It can be seen in lane 5 that only the methanol extract *V. pinetorum* (250 μg) has no significant effect on DNA cleavage protection (Figure 6.). 

## Conclusions

Present study showed that the acetone and methanol extracts of *V. pinetorum *shows strong antioxidant activity in the all activity assays namely β-carotene-linoleic acid test system, DPPH free radical scavenging, ABTS cation radical decolorisation and cupric reducing antioxidant capacity methods. Results reported in this study can be considered as the first detailed study on the phytochemical content and in-vitro antioxidant, anticholinesterase, antimicrobial activities and DNA damage protection effect of V. pinetorum. Further investigation should be done for isolation and identification of antioxidant active constituents of the extracts, especially acetone and methanol extract.
